# Efficacy of milrinone combined with norepinephrine in improving hemodynamics and protecting liver/kidney function in patients with septic shock

**DOI:** 10.12669/pjms.42.7.16206

**Published:** 2026-07

**Authors:** Cong Fu, Haoxuan Li, Jiaqi Ge, Xinxin Jiang, Lichen Guo

**Affiliations:** 1 Cong Fu, Department of Critical Care Medicine, Obstetrics & Gynecology Hospital of Fudan University, Shanghai Key Lab of Reproduction and Development, Shanghai Key Lab of Female Reproductive Endocrine Related Diseases, Shanghai 200433, P.R. China; 2 Haoxuan Li, Department of Critical Care Medicine, Obstetrics & Gynecology Hospital of Fudan University, Shanghai Key Lab of Reproduction and Development, Shanghai Key Lab of Female Reproductive Endocrine Related Diseases, Shanghai 200433, P.R. China; 3 Jiaqi Ge Department of Critical Care Medicine, The Affiliated Hospital of Jiangnan University, Xuxi, Jiangsu Province 214100, P.R. China; 4 Xinxin Jiang Department of Nephrology, Jing’an District Central Hospital, Shanghai 200000, P.R. China; 5 Lichen Guo, Department of Critical Care Medicine, Obstetrics & Gynecology Hospital of Fudan University, Shanghai Key Lab of Reproduction and Development, Shanghai Key Lab of Female Reproductive Endocrine Related Diseases, Shanghai 200433, P.R. China

**Keywords:** Milrinone, Norepinephrine, Septic shock, Hemodynamics, Organ function

## Abstract

**Objective:**

To investigate the effect of milrinone combined with norepinephrine in the treatment of septic shock.

**Methodology:**

Clinical data from one hundred patients with septic shock at the Affiliated Hospital of Jiangnan University between January 2022 and October 2025 were retrospectively collected. Patients treated with norepinephrine injection combined with milrinone composed the study group (n=53); patients who received norepinephrine injection alone were assigned to the control group (n=47). Hemodynamics, tissue perfusion, liver/kidney functions, prognosis, and adverse reactions were recorded in both groups.

**Results:**

At 72 hours after treatment, the mean arterial pressure (MAP), central venous pressure (CVP), and heart rate (HR) were better in the study group than in the control group (P<0.05). Serum levels of lactate (Lac), creatinine (Cr), blood urea nitrogen (BUN), alanine aminotransferase (ALT), and total bilirubin (TBIL) were significantly lower in the study group than in the control group (P<0.05). The lengths of intensive care unit (ICU) and total hospital stays in the study group were shorter than those in the control group, and 28-day mortality was lower (P<0.05). The incidence of adverse reactions in the study group was lower than that in the control group, but the difference was not statistically significant (P>0.05).

**Conclusion:**

Milrinone combined with norepinephrine can improve the hemodynamic status of patients with septic shock; reduce tissue hypoperfusion and liver and kidney dysfunction; and decrease 28 days mortality.

## INTRODUCTION

Septic shock, caused by an uncontrolled systemic inflammatory response and severe circulatory and cellular metabolic disorders induced by infection, is characterized by refractory hypotension, inadequate tissue perfusion, and multiple organ dysfunction.^1^,^2^ Epidemiological data show that the 28-day mortality of patients with septic shock remains as high as 30%–50%, even with standardized fluid resuscitation and anti-infective therapy.^3^–^5^

Norepinephrine, a first-line vasopressor recommended for septic shock, acts predominantly through α1-adrenergic receptor-mediated vasoconstriction, thereby increasing vascular tone and mean arterial pressure, while its β1-adrenergic activity may enhance myocardial contractility.^6^ It can rapidly correct hypotension and reduce complications such as pulmonary edema and tissue edema caused by excessive fluid resuscitation; thus, it plays an irreplaceable role in circulatory support.^7^,^8^ However, some patients respond poorly to norepinephrine and require high-dose maintenance, which may lead to excessive peripheral vasoconstriction, deteriorated microcirculatory perfusion, increased myocardial oxygen consumption, and an elevated risk of arrhythmias, ultimately leading to a failure to fundamentally reverse organ dysfunction.^9^ Previous studies have shown that compared with single-dose norepinephrine, norepinephrine combined with low-dose terlipressin (TP, a synthetic long-acting vasopressin analog) improves hemodynamic stability, decreases norepinephrine dependence, and supports multiorgan function recovery. Moreover, the combination of norepinephrine and TP provides a better safety profile in patients with septic shock but does not significantly impact 28 days mortality.^10^

Milrinone is a phosphodiesterase III inhibitor that increases the intracellular calcium concentration in cardiomyocytes by inhibiting the degradation of cyclic adenosine monophosphate, which has a β-receptor-independent positive inotropic effect.^11^ Milrinone exerts its inotropic and vasodilatory effects by inhibiting phosphodiesterase-3, thereby increasing intracellular cyclic AMP levels, inducing peripheral and pulmonary vasodilation, reducing cardiac preload and afterload, and enhancing myocardial contractility.^12^ Thus, milrinone treatment is associated with improved cardiac output and optimized tissue perfusion.^13^ Unlike β-adrenergic agonists, milrinone does not rely directly on β-receptor stimulation, making it less prone to β-receptor desensitization and potentially advantageous in patients with septic shock complicated by myocardial depression.^14^ Nevertheless, the vasodilatory effect of milrinone may cause further hypotension, and its single use carries a risk of circulatory instability, which limits its use alone in the hypotensive phase of shock.^15^,^16^

Therefore, we hypothesized that combining NE and milrinone might have synergistic advantages: NE maintains vascular tone and perfusion pressure to offset the hypotensive side effects of milrinone, and milrinone improves myocardial contractility, reduces afterload, increases cardiac output, and improves microcirculation. In this study, the clinical effects of single norepinephrine treatment and a combination of norepinephrine and milrinone were compared to evaluate differences in efficacy and safety in patients with septic shock. These results may provide clinical evidence and theoretical support for the individualized selection of vasoactive drugs and precise treatment of septic shock.

## METHODOLOGY

The clinical data of one hundred patients with septic shock admitted to the intensive care unit (ICU) of the Affiliated Hospital of Jiangnan University from January 2022 to October 2025 were retrospectively collected. Patients were divided into a study group (53 cases) and a control group (47 cases) according to the actual clinical medication regimen: the study group received a combination of norepinephrine and milrinone, whereas the control group received only a norepinephrine injection.

### Ethical approval:

The ethics committee of our hospital approved this study (number LS2025286; date: September 15, 2025).

### Inclusion criteria:

- The diagnostic criteria for septic shock were met according to the Sepsis-3 International Consensus Definitions.^17^
- Require vasoactive drugs to maintain blood pressure after fluid resuscitation.
- Complete clinical data;
- Age ≥ 18 years.

### Exclusion criteria:

- Complications included severe valvular heart disease, obstructive cardiomyopathy, acute myocardial infarction, or a history of malignant arrhythmia.
- Preexisting severe hepatic or renal failure, coagulation dysfunction, or immunodeficiency disease.
- Pregnant or lactating women.
- Hypersensitivity to milrinone, norepinephrine, or related drug components.
- Death, automatic discharge within 24 hours after admission, or missing data that could not be evaluated.
- Extreme poor short-term survival is expected because of brain death, advanced tumors, or other end-stage diseases.

### Treatment protocols:

After admission, both groups received conventional interventions, including rapid fluid resuscitation, broad-spectrum anti-infective therapy, oxygen therapy and respiratory support, blood glucose control, nutritional support, maintenance of internal environmental stability, and support for organ function.

In addition to conventional interventions, patients in the control group received norepinephrine via continuous intravenous infusion with a microinfusion pump. The initial dose ranged from 0.05–0.10 μg/(kg·min), and the infusion rate was adjusted in real time according to the patient’s mean arterial pressure (MAP), heart rate, and circulatory status to maintain a MAP ≥ 65 mmHg. The medication was administered continuously for 72 hours.

The study group received milrinone injections in addition to the norepinephrine regimen. A loading dose of milrinone at 50 μg/kg was administered via slow intravenous push (completed within 10 minutes), followed by continuous intravenous infusion at a maintenance dosage of milrinone at 0.375–0.750 μg/(kg ·minute). Blood pressure, heart rate, and other hemodynamic parameters were closely monitored during treatment, and the dose was adjusted on the basis of the patient’s tolerance. The medication was administered continuously for 72 hours.

### Observation indicators:

The following indicators were collected from all patients:

### Hemodynamic status:

Before treatment and at 72 hours after treatment, the mean arterial pressure (MAP), central venous pressure (CVP), and heart rate (HR) were recorded using an intensive care unit monitoring system and a pulse contour cardiac output monitor.

### Tissue perfusion and liver/kidney function:

Peripheral venous blood samples were collected on an empty stomach in the morning before treatment and 72 hours after treatment. After serum separation by centrifugation, blood lactate (Lac), creatinine (Cr), blood urea nitrogen (BUN), alanine aminotransferase (ALT), and total bilirubin (TBIL) levels were measured using an automatic biochemical analyzer.

### Clinical outcomes:

The lengths of ICU stay, total hospital stay, and 28-day mortality were recorded for both groups.

### Adverse reactions:

Continuous monitoring was performed during treatment, and the number of adverse reactions, such as arrhythmia, hypotension, myocardial ischemia, abnormal liver function, and thrombocytopenia, was recorded.

### Statistical analysis:

Data were analyzed using SPSS 25.0. A normality test was performed for numerical data, which are presented as the mean ±s when they were normally distributed. The differences in numerical data between the two groups were analyzed using the independent samples t test. Categorical variables are described as frequencies and constituent ratios (%) and were analyzed using the chi-square test or Fisher’s exact test. A P value < 0.05 was considered to indicate statistical significance.

## RESULTS

There were no significant differences in baseline data, including sex, age, body mass index (BMI), source of infection, duration of disease, APACHE II score, or underlying comorbidities, between the two groups (P > 0.05; Table-I). Before treatment, there were no significant differences in MAP, CVP, or HR between the two groups (P > 0.05). 72 hours after treatment, the MAP and CVP were greater in both groups than before treatment, while the HR was lower. The study group outperformed the control group (P < 0.05; Table-II).

**Table I:** Comparison of general data between the two groups.

Item	Study group (n=53)	Control group (n=47)	t/χ^2^value	P value
Age (*x̄*±*s*, years)	56.74±7.36	55.82±7.51	0.618	0.538
***Gender [n(%)]***				
Male	28(52.83)	26(55.32)	0.062	0.803
Female	25(47.17)	21(44.68)		
***BMI (*x̄*±*s*, kg/m²)***	22.37±2.08	21.62±2.15	1.771	0.080
Source of infection[n(%)]				
Pulmonary infection	31(58.49)	29(61.70)	0.294	0.961
Intra-abdominal infection	12(22.64)	11(23.40)		
Urinary system infection	6(11.32)	4(8.51)		
Bloodstream infection	4(7.55)	3(6.38)		
Time from onset to hospitalization (*x̄*±*s*, h)	3.31±1.09	3.26±1.12	0.226	0.822
APACHE II score (*x̄*±*s*, points)	21.68±3.74	21.13±3.69	0.739	0.462
***Underlying diseases***				
Hypertension	21(39.62)	17(36.17)	0.126	0.723
Hyperlipidemia	15(28.30)	14(29.79)	0.027	0.871
Diabetes mellitus	13(24.53)	10(21.28)	0.149	0.700
Chronic obstructive pulmonary disease	7(13.21)	3(6.38)	1.289	0.256
Other	6(11.32)	5(10.64)	0.011	0.913

**Table II:** Comparison of the hemodynamic status between the two groups (*x̄*±*s*).

Time	Group	n	MAP (mmHg)	CVP (cmH_2_O)	HR (beats/min)
Before treatment	Study group	53	54.28±4.16	6.14±1.21	124.42±9.46
	Control group	47	53.89±5.02	6.08±1.19	122.61±8.79
	*t value*		0.425	0.249	0.987
	*P value*		0.672	0.804	0.326
72 hours after treatment	Study group	53	70.45±5.59	10.36±1.98	90.94±6.41
	Control group	47	65.18±4.76	8.71±1.62	96.98±7.12
	*t value*		5.042	4.525	4.464
	*P value*		<0.001	<0.001	<0.001

Both groups had comparable pretreatment blood levels of Lac, Cr, BUN, ALT, and TBIL (P > 0.05). At 72 hours after treatment, the levels of Lac, Cr, BUN, ALT, and TBIL decreased and were significantly lower in the study group than in the control group (P < 0.05; Table-III).

**Table III:** Comparison of tissue perfusion and liver/kidney function between the two groups (*x̄*±*s*).

Time	Group	n	Lac (mmol/L)	Cr (umol/L)	BUN (mmol/L)	ALT (U/L)	TBIL (umol/L)
Before treatment	Study group	53	4.82±1.10	134.86±28.21	10.64±2.65	78.16±15.34	28.64±5.27
	Control group	47	4.75±1.16	136.12±30.72	11.04±2.42	76.91±16.76	29.31±4.96
	*t* value		0.310	0.306	0.785	0.389	0.652
	P value		0.758	0.760	0.435	0.698	0.516
72 hours after treatment	Study group	53	1.66±0.54	88.92±16.07	6.62±1.41	41.85±10.02	16.39±3.62
	Control group	47	2.40±0.65	99.39±19.86	8.36±1.68	50.01±12.43	20.47±4.15
	*t* value		6.216	2.911	5.630	3.631	5.380
	*P* value		<0.001	0.005	<0.001	0.001	<0.001

The ICU and total hospital stays in the study group were shorter than those in the control group. Similarly, the 28 days mortality rate (9.43%) was lower than that in the control group (25.53%) (P < 0.05; Table-IV). The incidence of adverse reactions was determined. There were nine adverse reactions in the control group, including arrhythmia (n=3), hypotension (n=2), myocardial ischemia (n=2), and abnormal liver function (n=2). In the study group, 4 adverse reactions, namely, arrhythmia (n=1), hypotension (n=1), and abnormal liver function (n=2), were recorded. A chi-square test revealed that the incidence of adverse reactions did not significantly differ between the two groups (p=0.085; Table-V).

**Table IV:** Comparison of Clinical Prognosis between the Two Groups.

Group	No. of Cases	ICU Length of Stay(d)	Total Hospital Stay(d)	28-day Mortality[n(%)]
Study Group	53	7.21±2.09	13.42±3.18	5(9.43)
Control Group	47	9.47±2.91	16.01±4.66	12(25.53)
*χ^2^/t* value		4.497	3.277	4.575
*P* value		<0.001	0.002	0.032

**Table-V:** Comparison of adverse reactions between the two groups [n(%)].

Group	No. of Cases	Arrhythmia	Hypotension	Myocardial Ischemia	Abnormal Liver Function	Total Incidence
Study Group	53	1(1.89)	1(1.89)	0(0.00)	2(3.77)	4(7.55)
Control Group	47	3(6.38)	2(4.26)	2(4.26)	2(4.26)	9(19.15)
*χ^2^value*						2.965
*P value*						0.085

## DISCUSSION

In this study, the clinical data of one hundred patients with septic shock were retrospectively analyzed to compare the clinical efficacy of milrinone combined with norepinephrine versus that of norepinephrine alone. The results confirmed that the combined regimen could significantly improve the patient’s hemodynamic status, reduce liver and kidney injuries, shorten the hospital stay, and decrease 28-day mortality but did not increase the risk of adverse reactions, providing reliable practical evidence for the clinical optimization of treatment strategies for septic shock.

Septic shock is a critical illness with persistently high mortality rates.^18^ The fundamental pathophysiological mechanisms of septic shock involve excessive systemic inflammation, vasomotor dysfunction, myocardial suppression, and profound hypoperfusion, ultimately leading to multiorgan dysfunction.^19^ Therefore, prompt hemodynamic optimization and organ-protective strategies are essential for improving clinical outcomes.^20^

At 72 hours after treatment, both groups showed higher mean arterial pressure and central venous pressure and lower heart rate than their respective pretreatment values. At this time point, the study group had significantly higher MAP and CVP and a significantly lower HR than the control group. The improvement in each index in the study group was significantly greater than that in the control group (P < 0.05), which is consistent with the conclusions of previous clinical studies.^13^ Patients with septic shock often present with vascular hyporesponsiveness and myocardial depression. Simply relying on norepinephrine to increase blood pressure may lead to excessive peripheral vasoconstriction, which in turn aggravates microcirculatory disturbance.^21^ Milrinone can enhance myocardial contractility and dilate resistance vessels, reduce cardiac afterload, and increase cardiac output.^22^ Therefore, a combined milrinone and norepinephrine regimen can increase circulatory efficiency while maintaining adequate perfusion pressure. This strategy facilitates rapid correction of the shock state, stabilizes heart rate, and reduces cardiac workload, thereby promoting faster and more stable recovery of hemodynamic parameters. These effects provide an important mechanistic basis for the efficacy of the combined regimen in reversing shock.^23^

Liver and kidney dysfunction and tissue lactate accumulation are core indicators for evaluating the severity and therapeutic effect of septic shock.^24^ At 72 hours after treatment, blood lactate, creatinine, blood urea nitrogen, alanine aminotransferase, and total bilirubin levels were lower than their respective pretreatment values in both groups and were significantly lower in the study group than in the control group.. Under septic shock conditions, persistent tissue hypoperfusion, hypoxia, and an inflammatory cascade can directly induce injury to renal tubular epithelial cells and hepatocytes, resulting in abnormally elevated liver and kidney function indicators.^25^ Milrinone can dilate microvessels, improve tissue oxygen supply, alleviate ischemia-reperfusion injury, and reduce the intensity of the systemic inflammatory response, thereby alleviating structural damage to and dysfunction of the heart, liver, and kidney; significantly accelerating lactate clearance; and protecting organ function.^26^ The findings of this study clearly indicate that combining milrinone with vasoactive drugs can significantly increase liver/kidney protection, providing important support for blocking the progression of sepsis to multiple organ failure.

Compared with those in the control group, the ICU and total hospital stays in the study group were significantly shorter, and the 28 days mortality was significantly lower (P < 0.05). These results are highly consistent with those of previous reports.^27^ We speculate that the rapid stabilization of hemodynamics and timely protection of liver and kidney function can significantly shorten the duration of circulatory support and liver and kidney function recovery; reduce the dependence on adjuvant support, such as mechanical ventilation and continuous renal replacement therapy; and lower the risk of disease deterioration and severe complications, thereby shortening the hospital stay, reducing medical burden, and ultimately decreasing 28 days mortality. As a first-line vasoactive drug recommended by international guidelines for septic shock, norepinephrine can rapidly increase peripheral vascular resistance by stimulating α-receptors and maintaining the perfusion pressure of vital organs.^28^

The ability of norepinephrine monotherapy to improve myocardial contractility and microcirculatory perfusion is limited, and its resuscitative efficacy is often suboptimal.^13^ Strategies have been developed to overcome this limitation of norepinephrine by combining it with other drugs.^29^-^31^ For example, the combination of norepinephrine and low-dose TP improved the hemodynamic indices and survival rate of patients with septic shock. The underlying mechanisms might be associated with reduced levels of inflammatory responses and improved renal function.^31^ The combination of norepinephrine and esmolol also improved cardiac function, hemodynamic parameters, and the inflammatory response, thus improving the prognosis of patients with septic shock.^29^

Milrinone is a phosphodiesterase III inhibitor that prevents the degradation of intracellular cyclic adenosine monophosphate (cAMP), thereby increasing cAMP levels in cardiomyocytes and vascular smooth muscle cells.^32^ As a result, it enhances myocardial contractility, induces peripheral vasodilation, and improves ventricular diastolic function. When combined with norepinephrine, which primarily restores vascular tone and maintains perfusion pressure, these complementary mechanisms may provide a more balanced and optimized therapeutic strategy for circulatory resuscitation in patients with septic shock.^33^ The results of this study suggest that milrinone combined with norepinephrine can block the pathophysiological progression of septic shock, with important clinical significance for improving long-term prognosis.

In terms of safety, this study suggested that the total incidence of adverse reactions was comparable between the two groups. Although milrinone has vasodilatory effects, combining it with norepinephrine can balance vasomotor effects and prevent drastic fluctuations in blood pressure. The results of this study confirm that the addition of milrinone to conventional treatment, under standardized dose control and strict hemodynamic monitoring, does not increase the risk of adverse reactions, such as arrhythmia, myocardial ischemia, or abnormal liver function. The combined regimen is thus safe and reliable for circulatory support in patients with septic shock.

### Strengths:

This retrospective analysis suggested that combining milrinone and norepinephrine has significant protective effects against septic shock. This novel strategy mitigated liver/kidney dysfunctions, improved hemodynamic stability, and reduced the length of hospital stay and 28 days mortality of patients with septic shock.

### Limitations

As a single-center retrospective analysis with a relatively small sample size, potential selection bias cannot be excluded. In addition, the absence of long-term follow-up limits the evaluation of sustained clinical outcomes and quality of life. Furthermore, differences in the individualized efficacy of milrinone among patients with varying degrees of myocardial dysfunction and shock severity have not been thoroughly explored.

## CONCLUSIONS

Milrinone combined with norepinephrine is safe and can significantly improve the hemodynamic status of patients with septic shock, effectively alleviating insufficient tissue perfusion and liver/kidney dysfunction, shortening the hospital stay, and reducing 28 days mortality.

### Recommendations:

Future large-scale, multicenter, prospective randomized controlled trials should further validate the long-term efficacy and safety of the combined regimen and investigate its underlying molecular mechanisms, including effects on microcirculation, inflammatory responses, and organ function protection. Such studies provide higher-level evidence to support the precise and individualized management of septic shock.

### Author`s Contributions:

**CF and HL:** Literature search, study design and manuscript writing.

**JG, XJ and LG:** Data collection, data analysis and interpretation. Critical Review.

**CF and HL:** Manuscript revision and validation and is responsible for the integrity of the study.

All the authors have read and approved the final manuscript.
